# An in vitro method to survey DNA methylation in peripheral blood mononuclear cells (PBMCs) treated by airborne particulate matter (PM_10_)

**DOI:** 10.1016/j.mex.2018.11.008

**Published:** 2018-11-17

**Authors:** Maryam Faraji, Zahra Pourpak, Kazem Naddafi, Ramin Nabizadeh Nodehi, Mohammad Hossein Nicknam, Mansour Shamsipour, Zahra Alizadeh, Soheila Rezaei, Narjes Soleimanifar, Alireza Mesdaghinia

**Affiliations:** a Department of Environmental Health Engineering, School of Public Health, Tehran University of Medical Sciences, Tehran, Iran; b Immunology, Asthma and Allergy Research Institute, Tehran University of Medical Sciences, Tehran, Iran; c Center for Air Pollution Research (CAPR), Institute for Environmental Research (IER), Tehran University of Medical Sciences, Tehran, Iran; d Molecular Immunology Research Center, Tehran University of Medical Sciences, Tehran, Iran; e Department of Research Methodology and Data Analysis, Institute for Environmental Research, Tehran University of Medical Sciences, Tehran, Iran; f Social Determinants of Health Research Center, Yasuj University of Medical Sciences, Yasuj, Iran

**Keywords:** Effect of airborne particulate matter on DNA methylation, PM_10_ (PM with aerodynamic diameter ≤10 μm), Epigenetic, 5-Methylscytosine (5-mC), 5-Hydroxymethylscytosine (5-hmC), In vitro DNA methylation, Air pollution

## Abstract

The International Agency for Research on Cancer (IARC) has defined outdoor air pollution and PM as the human carcinogen (Group 1), which mainly cause an increased risk of lung cancer. Scientists have considered epigenetic modifications as a possible mechanism to deal with adverse effects of air pollution. This study aimed to compare the effect of airborne PM_10_ (PM with aerodynamic diameter ≤10 μm) on in vitro global methylation in human peripheral blood mononuclear cells (PBMCs). PM_10_ was sampled in metropolitan Tehran, the capital of Iran. PBMCs were extracted from whole blood of healthy males and treated with PM_10_ suspension at concentrations of 50–300 μg/mL for 4 h. Untreated cells were used as the negative control. Genomic DNA was extracted from each sample using the DNA blood mini kit according to the manufacturer’s instruction. Moreover, 5-methylsytosine (%5-mC) and 5-hydroxymethylcytosine (%5-hmC) were measured by the enzyme-linked immunosorbent assay (ELISA) method. %5-mC and %5-hmC in each sample was compared with negative control and reported as difference %5-mC and %5-hmC.

## Graphical abstract

**Figure:**
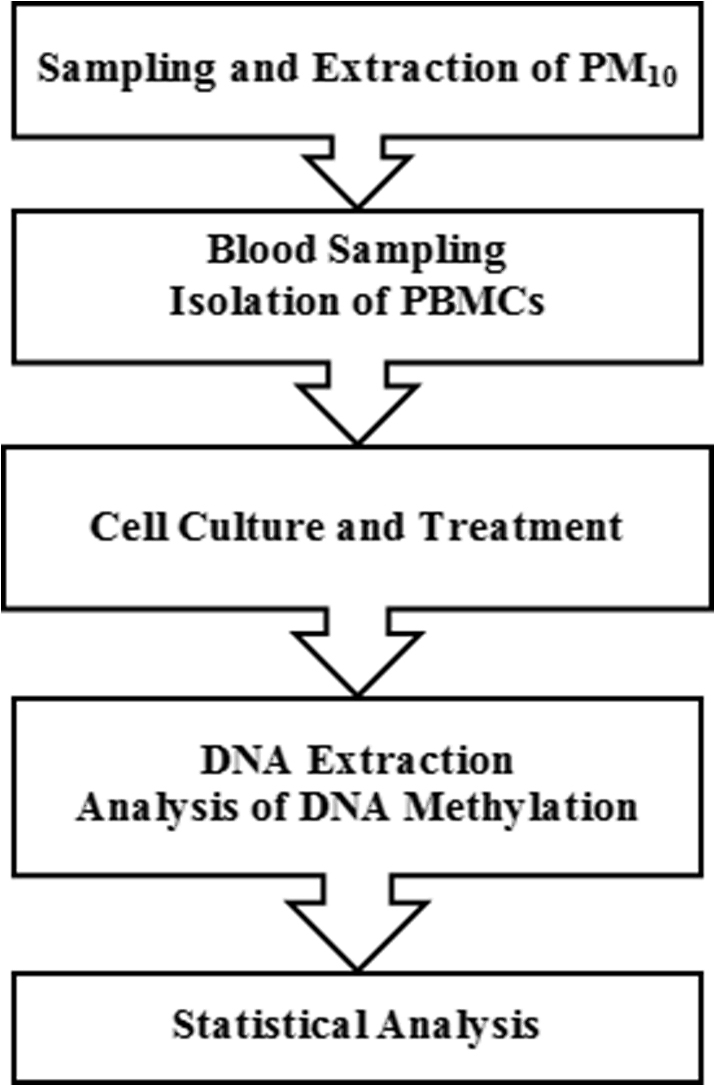

**Specifications Table**

**Table:** 

Subject Area	Select one of the following subject areas: • Environmental Science
More specific subject area:	Epigenetic in environmental science
Method name:	Effect of airborne particulate matter on DNA methylation
Name and reference of original method	***For DNA extraction see***: https://www.qiagen.com/us/shop//sample-technologies/dna/qiaamp-dna-mini-kit/#resources **For analysis of 5-mC see:** https://www.zymoresearch.eu/5-mc-dna-elisa-kits For analysis of 5-hmC see: https://www.zymoresearch.eu/quest-5-hmc-dna-elisa-kits
Resource availability	NA

## Method

**Fig. 1:**
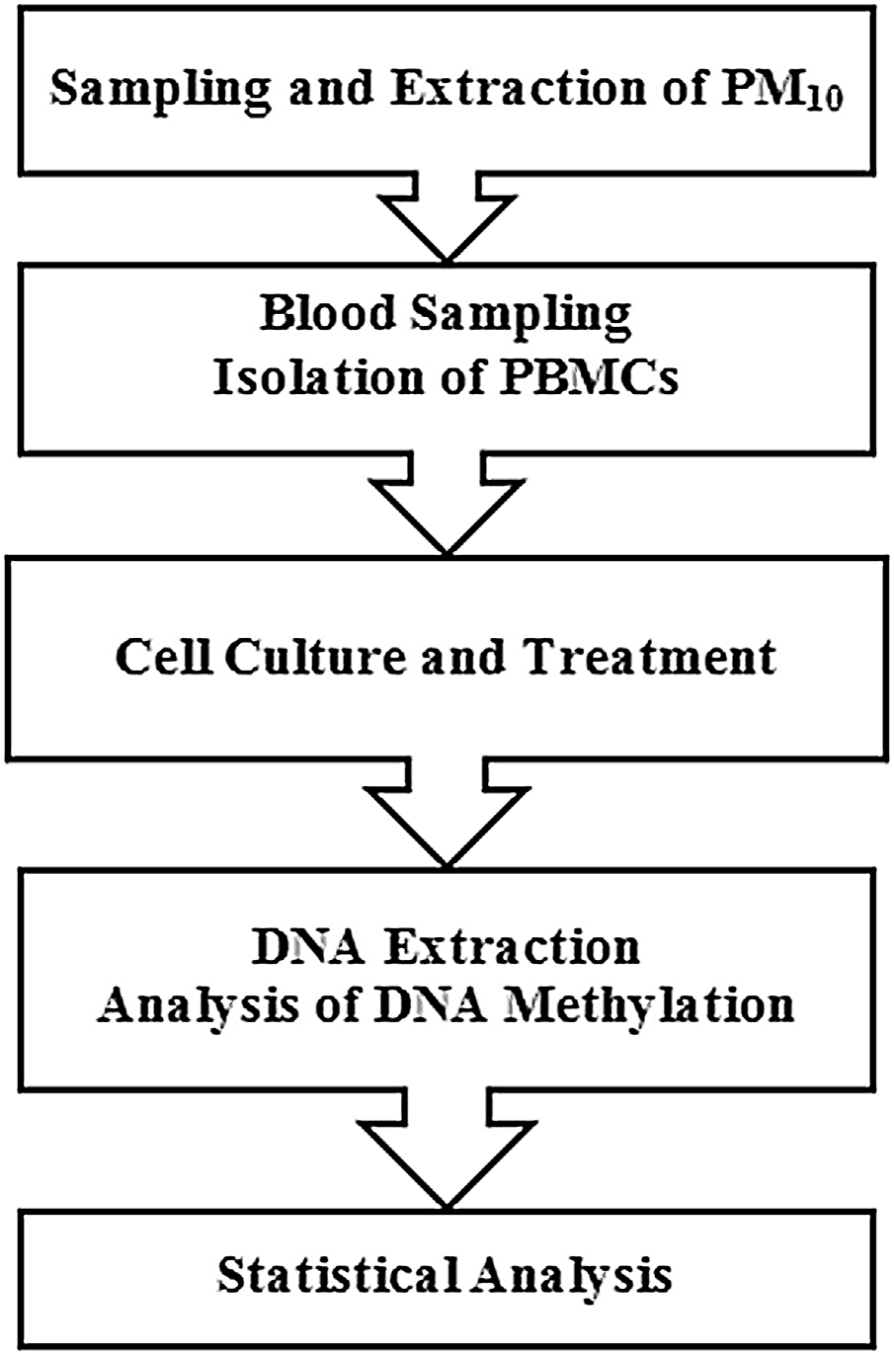
Flow diagram of the study stages.

The study stages were as follows: PM_10_ sampling; preparation of PM_10_ suspension; blood sampling and isolation of human peripheral blood mononuclear cells (PBMCs); cell culture and treatment; DNA extraction and analysis of DNA methylation and statistical analysis. All the stages are exhibited in Fig. 1.

## PM_10_ sampling

**Fig. 2:**
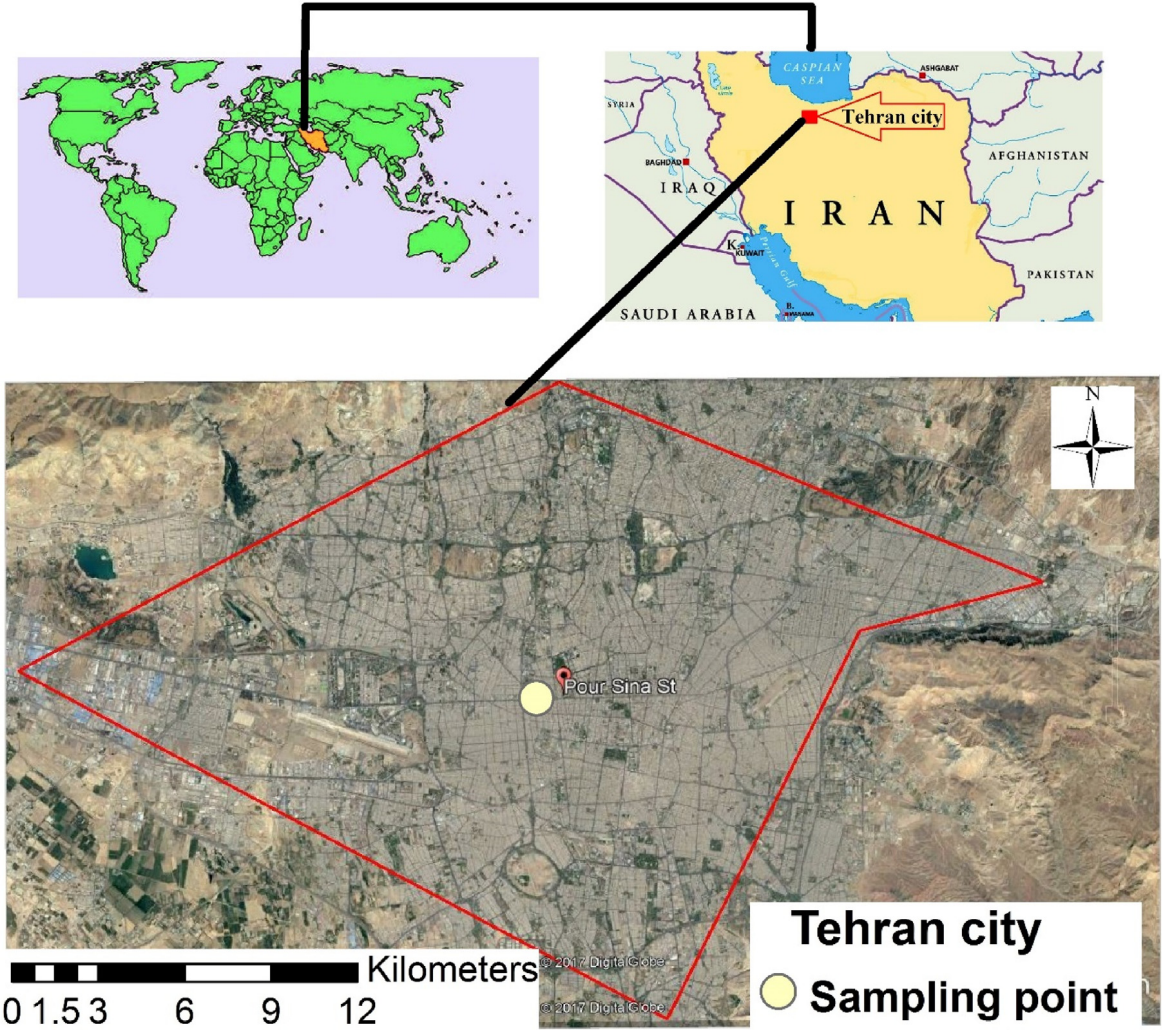
Map of the study area and PM_10_ sampling point.

Sampling was described in detail in our previous work [1]. Briefly, PM_10_ was sampled in Tehran, Iran (35°70′66.00″N, 51°39′38.55″E) (Fig. 2). Sampling was done as 24 h using a high-volume sampler (1.3–1.7 m^3^/min) (Grasebey, USA) included fiberglass filter (8 × 10 inch, grade G 653 Whatman, USA). Firstly, filters were heated for 2 h at 180 °C and maintained in a dark and dry place. Then, they were weighed with an analytical balance (±10 mg) before and after sampling to calculate the sampled PM mass. Weather information was obtained from a local meteorological monitoring station (Mehrabad).

## Preparation of PM_10_ suspension

**Fig. 3:**
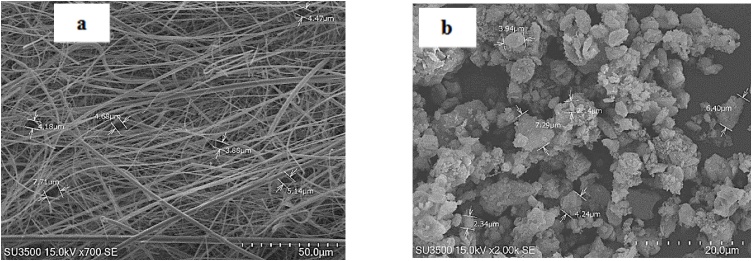
SEM images of the blank filter (a) and extracted PM_10_ (b).

Particle extraction for biological assay was done as the dry ultrasonic method [2]. Firstly, filters were cut into the small pieces and placed in 50 ml centrifuge tubes. Tubes were placed in an ultrasonic bath (Elma-ultrasonic, Germany) for 45 min followed by smooth sweeping with a brush. PM_10_ was collected into endotoxin-free vials, weighted and then stored at −18 °C until their use [[3], [4], [5]]. In the dry extraction method, presence of fiberglass fibers in PM samples is negligible and cannot cause noticeable biologic impacts on cells [5]. Morphological structure of blank filter and extracted samples were investigated by using a scanning electron microscope (SEM) (HITACHI, SU3500, Japan) (Fig. 3). Fiberglass fibers in the blank filter (Fig. 3a) were not observed in the extracted particles (Fig. 3b). Therefore, it can be concluded that dry extraction method used in this study prevented contamination of the PM_10_ samples with fiberglass fibers. The presence of fibers in the extracted samples is serious for biological assay because these minerals have their own inherent toxicity [6]. Immediately before use, extracted particles were autoclaved and suspended in RPMI-1640 culture medium to prepare PM_10_ suspension at different concentrations.

## Blood sampling and isolation of peripheral blood mononuclear cells (PBMCs)

This study was approved by the ethics committee of the Tehran University of Medical Sciences (IR.TUMS.SPH.REC.1395.841). Written informed consent was obtained from volunteers and their parents before initiating the study.

PBMCs were isolated from 20 mL whole blood sample from five males (16–18 years old), that enrolled in this study according to defined inclusion criteria, by density centrifugation on Ficoll-Hypaque gradient. Briefly, whole blood was diluted using 40 mL Ca^2+^/Mg^2+^ -free PBS (Biosera, France) in a laminar flux hood and gently mixed. Then, 30 mL Ficoll-Hypaque solution (Biosera, France) was added into the diluted blood and isolated PBMCs by density centrifugation (22 min, 2000 rpm, no acceleration, no brake). Layer of PBMCs was collected and washed by lysis buffer and isolation buffer and again centrifuged (400 g, 14 min, acceleration 6, brake 4). Next, PBMCs were suspended in 1 mL complete RPMI-1640 culture medium (Gibco BRL, San Diego, CA) and counted by trypan blue exclusion [7]. The viability of the cells was over 95%. Subsequently, the cells were maintained in a humidified incubator at 37 °C with 5% (v/v) CO_2_ in air.

## Cell cultures and treatment

PBMCs were cultured at a density of 500,000 cells/mL in RPMI-1640 culture medium for 24 h before treatment with PM suspension in an incubator at 37 °C with 5% (v/v) CO_2_. Further, cells from each donor were separately treated during 4 h with PM_10_ suspension at six concentrations of 50, 100, 150, 200, 250, and 300 μg/mL. The untreated cells were used as negative control in all the experiments. All the experiments at six concentrations were conducted in triplicate to confirm repeatability of results. After 4 h, the culture medium was collected and centrifuged at 5000 rpm for 8 min to separate cells from the supernatant. Finally, the cells were stored at −20 °C until DNA extraction [1].

## DNA extraction and analysis of DNA methylation

Genomic DNA was extracted from each sample using the QIAamp DNA blood mini kit (Qiagen, Hilden, Germany) according to the manufacturer’s instruction. DNA concentration was measured based on absorbance at 260 nm using Nanodrop spectrophotometer (NanoDrop Technologies, Wilmington, DE, USA).

Global %5-mC and %5-hmC in the extracted DNA samples were measured by the enzyme-linked immunosorbent assay (ELISA) method with 5-mC and Quest 5-hmC™ DNA ELISA kits (both from Zymo Research, Orange, CA, USA) using 100 ng extracted genomic DNA according to the manufacturers’ protocols. All samples were assayed in duplicate. The detection limit per 100 ng single-stranded DNA was 0.5% for 5-mC and 0.02% for 5-hmC [1].

## Statistical analysis

PM_10_ concentrations in the methylation test at six levels (50, 100, 150, 200, 250 and 300 μg/mL) were considered as independent variable. The effect of PM_10_ concentration on the difference of %5-mC or %5-hmC (change of %5-mC and %5-hmC in the treated cells in respect to the negative control) as the response variables was investigated using R software version 3.4.3 [8,9]. Significant level was defined as p-value lower than 0.05. According to the Shapiro–Wilk test for the 5-mC difference (p = 0.01), the effect of PM_10_ concentration on the value of 5-mC was investigated using the non-parametric test of Scheirer Ray Hare in Fisheries Stock Analysis (FSA) package [10] and the results were reported as median ± interquartile range (IQR). Normality and homogeneity of the 5-hmC difference were approved considering the p-value of concentration 0.11 in the Shapiro–Wilk test and 0.89 in the Bartlett test. Thus, analysis of variance (ANOVA) test was applied to compare the 5-hmC average. Moreover, Dunn test and TukeyHSD were respectively selected as post-hoc tests to assess the 5-mC and 5-hmC average between different PM_10_ concentrations.

## Conflict of interest

The authors declare that they have no competing financial interests.
